# Lake Baikal amphipods and their genomes, great and small

**DOI:** 10.18699/vjgb-24-36

**Published:** 2024-06

**Authors:** P.B. Drozdova, E.V. Madyarova, A.N. Gurkov, A.E. Saranchina, E.V. Romanova, J.V. Petunina, T.E. Peretolchina, D.Y. Sherbakov, M.A. Timofeyev

**Affiliations:** Irkutsk State University, Irkutsk, Russia Baikal Research Centre, Irkutsk, Russia; Irkutsk State University, Irkutsk, Russia; Irkutsk State University, Irkutsk, Russia Baikal Research Centre, Irkutsk, Russia; Irkutsk State University, Irkutsk, Russia; Limnological Institute of the Siberian Branch of the Russian Academy of Sciences, Irkutsk, Russia; Limnological Institute of the Siberian Branch of the Russian Academy of Sciences, Irkutsk, Russia; Limnological Institute of the Siberian Branch of the Russian Academy of Sciences, Irkutsk, Russia; Irkutsk State University, Irkutsk, Russia Limnological Institute of the Siberian Branch of the Russian Academy of Sciences, Irkutsk, Russia Novosibirsk State University, Novosibirsk, Russia; Irkutsk State University, Irkutsk, Russia

**Keywords:** Lake Baikal, amphipods, species flocks, speciation, population genetics, genomics, озеро Байкал, бокоплавы, букеты видов, видообразование, генетика популяций, геномика

## Abstract

Endemic amphipods (Crustacea: Amphipoda) of Lake Baikal represent an outstanding example of large species flocks occupying a wide range of ecological niches and originating from a handful of ancestor species. Their development took place at a restricted territory and is thus open for comprehensive research. Such examples provide unique opportunities for studying behavioral, anatomic, or physiological adaptations in multiple combinations of environmental conditions and thus attract considerable attention. The existing taxonomies of this group list over 350 species and subspecies, which, according to the molecular phylogenetic studies of marker genes, full transcriptomes and mitochondrial genomes, originated from at least two introductions into the lake. The studies of allozymes and marker genes have revealed a significant cryptic diversity in Baikal amphipods, as well as a large variance in genetic diversity within some morphological species. Crossing experiments conducted so far for two morphological species suggest that the differences in the mitochondrial marker (cytochrome c oxidase subunit I gene) can potentially be applied for making predictions about reproductive isolation. For about one-tenth of the Baikal amphipod species, nuclear genome sizes and chromosome numbers are known. While genome sizes vary within one order of magnitude, the karyotypes are relatively stable (2n = 52 for most species studied). Moreover, analysis of the diversity of repeated sequences in nuclear genomes showed significant between-species differences. Studies of mitochondrial genomes revealed some unusual features, such as variation in length and gene order, as well as duplications of tRNA genes, some of which also underwent remolding (change in anticodon specificity due to point mutations). The next important steps should be (i) the assembly of whole genomes for different species of Baikal amphipods, which is at the moment hampered by complicated genome structures with high repeat content, and (ii) updating species taxonomy taking into account all the data.

## Abstract (ru)

Эндемичные амфиподы (Crustacea: Amphipoda) озера Байкал – это один из наиболее ярких примеров возникновения большого количества видов (так называемых букетов видов), занимающих разнообразные экологические ниши, от небольшого числа исходных видов, которое происходило на ограниченной территории и потому доступно для всестороннего исследования. Подобные примеры предоставляют уникальные возможности изучения поведенческих, анатомических и физиологических адаптаций во множестве комбинаций условий среды и потому привлекают большое внимание. Существующие варианты таксономической классификации этой группы насчитывают более 350 морфологических видов и подвидов, которые, согласно молекулярно-филогенетическим исследованиям маркерных генов, полных транскриптомов и митохондриальных геномов, произошли в результате не менее двух вселений в озеро. Исследования изоферментов и маркерных генов выявили существенное криптическое разнообразие байкальских амфипод, а также существенный разброс по уровню генетического разнообразия внутри некоторых морфологических видов. Экспериментальная проверка, проведенная на данный момент только для двух морфологических видов, показывает возможную применимость митохондриального маркера, гена первой субъединицы цитохром c-оксидазы, для предсказания репродуктивной изоляции. Приблизительно у десятой части видов байкальских амфипод был изучен размер ядерного генома и хромосомные числа, что позволило выявить почти десятикратную вариабельность размера генома при стабильных (2n = 52 для большинства изученных видов) кариотипах. При этом анализ разнообразия повторов в ядерных геномах показал существенные межвидовые различия. Кроме того, выявлены необычные особенности некоторых митохондриальных геномов, такие как вариабельность по длине и по порядку генов, а также дупликации генов тРНК, часть из которых подверглась ремолдингу (изменению специфичности антикодона за счет точечных мутаций). Следующими важными шагами должны стать сборка полных геномов для разных видов байкальских амфипод, чему на данном этапе препятствует сложная структура этих геномов с большим содержанием повторов, и обновление таксономической классификации видов с учетом комплекса полученных данных.

## Introduction

Ancient lakes are known speciation hotspots. However, even against this background, the biodiversity of Lake Baikal, the age of which is estimated as 25–30 or even 70 million years, stands out (Cristescu et al., 2010; Mats et al., 2011). The representatives of the order Amphipoda (Crustacea) constitute one of the largest groups of closely related species found in Baikal.

The diversity of amphipods in Baikal may be partially attributed to the broad range of habitats and ecological niches they occupy, as the species within this group differ in habitat depth (0–1,642 meters), feeding habits, and reproductive periods (Takhteev, 2000a, b). However, many species share the same habitat, being at the same time similar in size, feeding spectra and reproductive periods (Takhteev, 2000a, b), which raises the question of the evolutionary forces that drove their speciation. Earlier reviews have already presented global conclusions about the origin of Baikal endemic fauna based on molecular data from multiple studies (Sherbakov, 1999; Sherbakov et al., 2017). However, the recent years have seen the accumulation of a lot of new data, especially high-throughput sequencing, which have uncovered new details on speciation and genome evolution in Baikal amphipods.

## How many amphipod species are there in Baikal?

Morphological classification

Currently, the formal identification of Baikal amphipod species is based on the morphological criterium, i. e. the presence of a unique set of morphological traits in all studied individuals of a particular species. The number of morphological species and subspecies in Baikal exceeds 350 (Takhteev, 2000a; Kamaltynov, 2001; Takhteev et al., 2015). In the case of Baikal amphipods, subspecies were mostly derived from morphological varieties that differed less than species would (Bazikalova, 1945; Takhteev, 2000a). All these species belong to the phylum Arthropoda, subphylum Crustacea, class Malacostraca, order Amphipoda, and superfamily Gammaroidea (Sket et al., 2019). The numbers of subspecies, species, genera and families differ according to different authors (Takhteev, 2019), but the most evident discrepancies are attributed to differing taxonomic levels (subspecies/species, congeneric species/ different genera etc.).

Multiple classifications complicate studies in Baikal amphipods. From a practical point of view, the most important discrepancies for researchers are different generic names for the same species. The correspondence between the names suggested by different authors can be easily checked using the World Amphipoda Database (WAD; https://www.marine species.org/amphipoda/) (Horton et al., 2023). It is worth noting that the systematics accepted by WAD (Kamaltynov, 2001, 2009) does not have an associated identification key, and thus many manuscripts use the species names indicated in the existing keys. The most comprehensive key for Baikal amphipods is still (Bazikalova, 1945), although some groups are covered in more detail in later sources (Bazikalova, 1962; Takhteev, 2000a). The only available English identification key for the genera of Baikal amphipods is provided by (Sket et al., 2019). An English language checklist of all known species according to the same classification is compiled in (Takhteev et al., 2015). However, none of the sources include the species described after 2000: Eulimnogammarus messerschmidtii Bedulina et Takhteev, 2014 (Bedulina et al., 2014), Eulimnogammarus etingovae and Eulimnogammarus tchernykhi Moskalenko, Neretina < Yampolsky, 2020 (Moskalenko et al., 2020).

Molecular genetics approaches to classification

Molecular phylogenetic studies in Baikal amphipods revealed three important conclusions. First, all studied species cluster within the freshwater radiation of the morphological genus Gammarus Fabricius, 1775 at the phylogenetic tree, which provides evidence of their descent from Gammarus-like freshwater ancestors (Macdonald III et al., 2005; Hou et al., 2014). Second, studies utilizing phylogenetic marker genes have shown that Baikal amphipods fall into two clades (Sherbakov, 1999; Macdonald III et al., 2005), indicating that their ancestors invaded the lake at least twice. This conclusion is supported by the phylogeny based on single-copy orthologs in transcriptomes (Naumenko et al., 2017) and whole mitochondrial genomes (Romanova et al., 2016a). The first invasion gave rise to a much smaller number of recent species than the second invasion (Bazikalova, 1945; Naumenko et al., 2017). Third, several species of Baikal amphipods were found to exhibit cryptic diversity, i. e. the presence of genetically distinct groups that are morphologically indistinguishable or hard to distinguish.

Studies of allozyme spectra showed significant (in many cases species-level) differences within morphological species and led to suggestions to elevate some subspecies to species rank (Yampolsky et al., 1994; Väinölä, Kamaltynov, 1999) or, vice versa, synonymize (Daneliya et al., 2009). The differences in allozyme frequencies may indicate the presence of isolated populations, but they are difficult to directly translate into species boundaries. This issue also affects the outcomes of phylogenetic marker sequencing, albeit to a lesser degree. In this case, species delimitation may rely on calculated threshold values of patristic distances (Lefébure et al., 2006) or other techniques that take into account genetic distances, phylogenetic tree topology or shared alleles (Fišer et al., 2018). However, the obtained sample clusters could not be safely assigned to biological species. Therefore, they are termed mo-lecular operational taxonomic units (MOTUs) (Blaxter, 2004).

Folmer fragment of the cytochrome c oxidase subunit I gene (COI or cox1) is the most well-known and frequently used marker sequence for amphipods and many other invertebrates (Folmer et al., 1994; Hebert et al., 2003). It is important to note that mitochondrial and nuclear-based phylogenies often produce conflicting results, which is known as mito-nuclear discordance (Toews, Brelsford, 2012). In order to draw reliable conclusions about separated genetic lineages, which would indicate reproductively isolated species, it is recommended to also employ nuclear markers. Popular nuclear markers include rRNA gene clusters as well as whole-genome markers such as ultraconserved elements (UCEs), restriction site-associated DNA (RADs), and single-copy orthologs (SCOs) (Eberle et al., 2020). From this list, SCOs have already been utilized to study Baikal amphipods (Naumenko et al., 2017; Drozdova et al., 2021); for other amphipods, RADs have also been used (Jordan et al., 2020; Weston et al., 2022; Eme et al., 2023).

Population genetic diversity

**Fig. 1.**
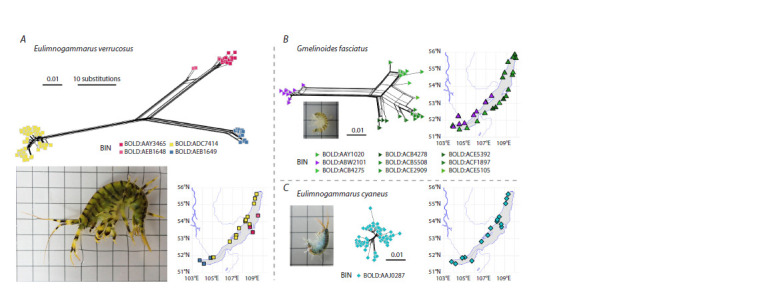
Comparison of the levels of population genetic diversity of the COI fragment within the best studied morphological species E. verrucosus (А), Gm. fasciatus (B) and E. cyaneus (C). Shown are representative photographs of each species at the same scale (grid size is 5 mm), along with split phylogenetic networks at the same scale (scale bar is 1 % substitutions, i. e. 5.1 substitutions in the 510-bp alignment), and corresponding sampling points. Sequence data were obtained from the BOLD database (Ratnasingham, Hebert, 2007). Sampling coordinates were added or corrected based on the original publications (Fazalova et al., 2010; Petunina, 2015; Romanova et al., 2016a; Gurkov et al., 2019). Different colors on networks and maps correspond to different barcode index numbers (BINs) automatically determined by BOLD (Ratnasingham, Hebert, 2013). For detailed methodology, please refer to https://github.com/drozdovapb/Baikal-amphipods-review-post-chr2023.

In total, intraspecies diversity has been studied using different methods and with varying geographical coverage for over 20 morphological species of Baikal amphipods (Supplementary Material 1)1. Some of these species showed substantial intraspecific diversity (Gomanenko et al., 2005; Daneliya et al., 2011; Gurkov et al., 2019). It is noteworthy that even species with comparable distribution and ecological characteristics can exhibit dramatic differences in the level of intraspecific diversity (Fig. 1). For example, it was found that the species Eulimnogammarus verrucosus (Gerstfeldt, 1858), common in the littoral zone, is actually composed of at least three genetic lineages, inhabiting the western (up to the source of the Angara river), southern and eastern parts of the Baikal shore (W, S, and E), respectively. Intraspecific pairwise differences in COI sequences reached 13 %, which is similar to the distances between morphological species (Gurkov et al., 2019). The most recent common ancestor of these lineages, according to a molecular clock-based estimate, existed around 4.5 million years ago (Drozdova et al., 2022). A nuclear marker, 18S rRNA gene fragment, fully corroborated this division (Gurkov et al., 2019).


Supplementary Materials are available in the online version of the paper: https://vavilovj-icg.ru/download/pict-2024-28/appx13.xlsx


Gmelinoides fasciatus (Stebbing, 1899) is another species common in the shallow water. It is also divided into genetic lineages correlated with geography, but here the differences are less pronounced, reaching about 8 % (Gomanenko et al., 2005), and the last common ancestor existed around 2 million years ago (Bukin et al., 2018). A nuclear marker, intron of the ATP synthase β subunit gene, showed a lower genetic diversity but also supported intraspecific differentiation (Kovalenkova, 2018). In contrast, preliminary data on the only pelagic planktonic species of Baikal amphipods, Macrohectopus branickii (Dybowsky, 1874), based on the fragments of the mitochondrial genes COI and NADH dehydrogenase fifth subunit (ND5 or nad5) (Petunina et al., 2023; Zaidykov et al., 2023) did not reveal geographically separated genetic lineages.

Finally, Eulimnogammarus cyaneus (Dybowsky, 1874), another widely distributed species inhabiting a significant part of the Lake Baikal littoral, exhibits very weak genetic differentiation based on the COI fragment (Gurkov et al., 2019) but much more pronounced differentiation according to allozyme data (Mashiko et al., 2000). Furthermore, it is important to note that the borders between genetic lineages of E. verrucosus, such as the Angara river outflow, do not hold for Gm. fasciatus (Fig. 1, А, B); the geographic barriers for Gm. fasciatus are unclear. The source of the Angara river started to form at most 120,000 years ago (Arzhannikov et al., 2018), thus being much younger that the last common ancestor of E. verrucosus populations dwelling at different sides of the outflow (3.81 million years ago) (Drozdova et al., 2022). The current cryptic diversity within E. verrucosus and Gm. fasciatus appears to reflect past distribution barriers, such as dwelling in refugia during non-favorable climatic conditions (Bukin et al., 2018).

Reproductive barriers and cryptic species

Reproductive isolation is crucial for biologically sensible species delimitation. However, this issue has just recently started to be explored for Baikal amphipods. To date, experimental checks for reproductive incompatibility have only been carried out for two widely distributed littoral species, E. verrucosus and E. cyaneus. Crossing experiments were conducted with representatives of populations from Listvyanka (W) and Port Baikal (S) for both species (these populations were chosen due to the closest geographic proximity of different genetic lineages), and also from Ust-Bargusin (E) for E. verrucosus. In the case of E. verrucosus, both prezygotic and postzygotic reproductive barriers were found. Although these barriers are not absolute, their combination can ensure reproductive isolation when different lineages are mixed. In the case of E. cyaneus, the analysis of representatives of the populations separated by the Angara river outflow did not show any prezygotic or postzygotic barriers. Mate choice was random, and upon crossing, at least the first generation hybrids developed normally (Drozdova et al., 2022, 2023). Therefore, in the case of E. verrucosus and E. cyaneus, differences in COI sequences indeed correlate with the presence of reproductive barriers. However, it would be premature to establish a general rule for Baikal amphipods based solely on these findings. It is necessary to conduct such experiments for other genera to draw comprehensive conclusions. Further research on reproductive barriers, as well as genomes and gene expression, may aid in comprehending the factors that contribute to reproductive incompatibility and thus serve as the genetic basis of speciation.

The next steps that need to be undertaken are renewal of the Baikal amphipod taxonomy and species redescription taking into account biological reality and possible competition between cryptic species. This necessity is not unique to Baikal, as cryptic species complexes without formal species descriptions are also characteristic of many other amphipods, including popular ecotoxicological models Gammarus fossarum and Hyalella azteca (Jourdan et al., 2023). However, it underlines the critical importance of always specifying the particular sampling place for Baikal amphipods in every publication and identifying the genetic lineage whenever possible.

## What is known about genomes of Lake Baikal amphipods?

The genetics of Baikal amphipods is a relatively understudied area, with most of the research focusing on individual genetic markers. Nuclear genome sizes have been estimated using cytogenetic methods such as Feulgen image analysis densitometry (FIAD) and flow cytometry (FCM) for 36 morphological species (Jeffery et al., 2017; Drozdova et al., 2022). Karyotypes have been studied for 35 morphological species (Salemaa, Kamaltynov, 1994; Kamaltynov, 2001; Natyaganova, Sitnikova, 2012; Barabanova et al., 2019) (Supplementary Material 2). Transcriptome sequencing data are available for over 60 morphological species (Naumenko et al., 2017; Drozdova et al., 2022), enabling the extraction of most protein-coding gene sequences, as well as partial or complete mitochondrial genomes. These transcriptome assemblies are particularly valuable for proteomic studies (Bedulina et al., 2021; Zolotovskaya et al., 2021). Genome DNA sequencing data are available for seven species, which enabled the assembly of mitochondrial genomes and can be used to evaluate the diversity of repeated sequences in nuclear genomes (Rivarola-Duarte et al., 2014; Romanova et al., 2016a, 2021; Rivarola-Duarte, 2021; Yuxiang et al., 2023) (Supplementary Material 3).

Genome size variation and its possible causes

**Fig. 2.**
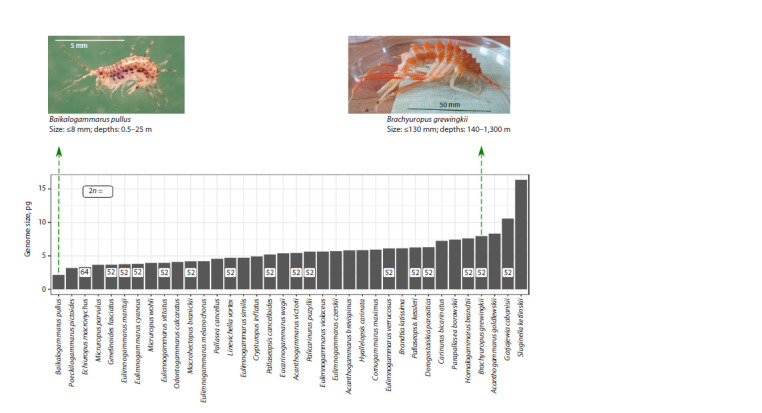
Nuclear genome sizes of Baikal amphipods, as estimated with FIAD (Jeffery et al., 2017), and their chromosome numbers (Salemaa, Kamaltynov, 1994; Kamaltynov, 2001; Natyaganova, Sitnikova, 2012). Please refer to Supplementary Material 2 for full data set. Species names are given according to (Jeffery et al., 2017). The photographs show Baikalogammarus pullus (Dybowsky, 1874), which has the smallest genome and small body length, and dwells in the littoral and sublittoral zones, and Brachyuropus grewingkii (Dybowsky, 1874), which is a deep-water species and one of the largest. The ecological characteristics of these species are given according to (Kamaltynov, 2001). The photo of B. grewingkii was generously provided by Ekaterina Shchapova.

The genome sizes of the studied Baikal amphipods varied from 2 to 17 pg (1 pg is approximately 1 Gb) (Jeffery et al., 2017) (Fig. 2), which falls within the known range of amphipod genome sizes (Hultgren et al., 2018). For up-to-date information on accumulated data one can refer to the Animal Genome Size Database (http://www.genomesize.com/) (Gregory et al., 2007).

When comparing data obtained using different methods, it is worth keeping in mind that crustacean genome size estimates obtained with FIAD are typically slightly lower than those obtained with FCM (Wyngaard et al., 2022). Notably, genome size differences accumulate quite rapidly, as evidenced by the differing genome sizes of E. verrucosus lineages (6.1 pg for the E, 6.9 pg for the W, and 8.0 pg for the S lineage) (Drozdova et al., 2022). The analysis of genome sizes in different species showed a weak positive correlation with both maximal body length and habitat depths, which corresponds to the known ecological trends (Jeffery et al., 2017). However, chromosome numbers were found to be identical (2n = 52) for 33 out of 35 studied species (Salemaa, Kamaltynov, 1994; Kamaltynov, 2001; Natyaganova, Sitnikova, 2012) (Fig. 2), which corresponds to the modal chromosome number for gammaroid amphipods (Coleman, 1994). The lack of correlation between chromosome numbers and genome sizes suggests that repeated sequences significantly contribute to this variation. Analysis of the diversity of repeated sequences revealed significant differences between species of Baikal amphipods (Yuxiang et al., 2023). In all studied species, the proportion of reads included in repeat clusters exceeded 50 % (Rivarola-Duarte et al., 2014; Yuxiang et al., 2023).

Mitochondrial genomes

The mitochondrial genome is the most extensively studied part of the genome in Baikal amphipods. It is a small, high-copy DNA molecule, and its sequence is generally easy to assemble from low-coverage genome-wide sequencing (Smith, 2016). Animal mitochondrial genomes are typically circular with a length of about 16 kb and contain 13 protein-coding genes, 2 rRNA genes and 22 tRNA genes. However, significant differences in genome architecture, size, and composition are known (Lavrov, Pett, 2016).

At the moment, eight complete and six partial mitochondrial genomes have been published for Baikal amphipods (Rivarola- Duarte et al., 2014; Romanova et al., 2016a–c, 2021; Mamos et al., 2021) (Supplementary Material 4). Most of these assemblies are within 15–18 kb in length, but the mitochondrial genome of M. branickii is over 42 kb-long, making it one of the largest known animal mitochondrial genomes (Romanova et al., 2021). Furthermore, mitochondrial genomes of some Baikal amphipods exhibit gene order rearrangements, gene duplications and the phenomenon of tRNA gene remolding, i. e. changes in tRNA specificity due to a mutation in the anticodon sequence. Remolding is not unique for Baikal amphipods but occurs with higher frequency than in other amphipods (Romanova et al., 2020).

## Perspectives in whole-genome studies

The next important step in the development of genome-wide studies of Baikal amphipods should be the assembly of whole nuclear genomes for a number of species. For the world amphipod fauna, seven genome assemblies are mentioned in the literature (Supplementary Material 5). Four of them (H. azteca, Trinorchestia longiramus, Platorchestia hallaensis, and Parhyale hawaensis) belong to the infraorder Talitrida (Kao et al., 2016; Poynton et al., 2018; Patra et al., 2020, 2021). Three species belong to the infraorder Gammarida (Gammarus lacustris, G. roeselii, and E. verrucosus). One of these species, E. verrucosus, inhabits Baikal (Jin et al., 2019; Cormier et al., 2021; Rivarola-Duarte, 2021). The genomes of gammarids are the largest within this list. Not surprisingly, creation of a high-quality assembly of these genomes is complicated and currently at the draft stage, with N50 of all assemblies being below 5 kb, and only the genome of G. roeselii being publicly available.

The development of third-generation genome sequencing techniques provides hope that technical difficulties in assembly of complex gammarid genomes can be overcome. For example, the assembly of the Antarctic krill, Euphausia superba, genome, which with 48 Gb is the largest assembled animal genome to date, demonstrates the potential of this technology (Shao et al., 2023). High-quality genome assemblies will greatly enhance the research on the adaptation mechanisms of endemic amphipods to various conditions in Lake Baikal and tracing their evolutionary history. This will be due to a wider range of possibilities for retrieving full gene sets (which is impossible with the current transcriptomic data) and regulatory elements, as well as new data on population history (Bourgeois, Warren, 2021) and higher resolution for phylogenetic analysis

## Conflict of interest

The authors declare no conflict of interest.

## References

Arzhannikov S.G., Ivanov A.V., Arzhannikova A.V., Demonterova E.I.,
Jansen J.D., Preusser F., Kamenetsky V.S., Kamenetsky M.B. Catastrophic
events in the Quaternary outflow history of Lake Baikal.
Earth-Sci. Rev. 2018;177:76-113. DOI 10.1016/j.earscirev.2017.
11.011

Barabanova L., Galkina S., Mikhailova E. Cytogenetic study on the invasive
species Gmelinoides fasciatus in the ecosystem of the Gulf of
Finland. J. Mar. Biol. Assoc. UK. 2019;99(3):611-618. DOI 10.1017/
S0025315417001357

Bazikalova A.Y. Amphipods of Lake Baikal. Trudy Baykal’skoy Limnologicheskoy
Stantsii = Proceedings of the Baikal Limnological Station.
1945;11:1-440 (in Russian)

Bazikalova A.Y. Taxonomy, ecology, and distribution of the genera
Micruropus
Stebbing and Pseudomicruropus nov. gen. (Amphipoda,
Gammaridea). Systematics and ecology of crustaceans of Baikal.
Trudy Limnologicheskogo Instituta = Proceedings of the Limnological
Institute. 1962;2(22):3-140 (in Russian)

Bedulina D.S., Takhteev V.V., Pogrebnyak S.G., Govorukhina E.B.,
Madyarova
E.V., Lubyaga Y.A., Vereshchagina K.P., Timofeyev
M.A., Luckenbach T. On Eulimnogammarus messerschmidtii,
sp. n. (Amphipoda: Gammaridea) from Lake Baikal, Siberia, with
redescription of E. cyanoides (Sowinsky) and remarks on taxonomy
of the genus Eulimnogammarus. Zootaxa. 2014;3838(5):518-544.
DOI 10.11646/zootaxa.3838.5.2

Bedulina D., Drozdova P., Gurkov A., von Bergen M., Stadler P.F.,
Luckenbach T., Timofeyev M., Kalkhof S. Proteomics reveals sexspecific
heat shock response of Baikal amphipod Eulimnogammarus
cyaneus. Sci. Total Environ. 2021;763:143008. DOI 10.1016/
j.scitotenv.2020.143008

Blaxter M.L. The promise of a DNA taxonomy. Philos. Trans. R. Soc.
Lond. B. Biol. Sci. 2004;359(1444):669-679. DOI 10.1098/rstb.
2003.1447

Bourgeois Y.X.C., Warren B.H. An overview of current population
genomics methods for the analysis of whole-genome resequencing
data in eukaryotes. Mol. Ecol. 2021;30(23):6036-6071. DOI
10.1111/mec.15989

Bukin Yu.S., Petunina J.V., Sherbakov D.Yu. The mechanisms for genetic
diversity of Baikal endemic amphipod Gmelinoides fasciatus:
relationships between the population processes and paleoclimatic
history of the Lake. Russ. J. Genet. 2018;54(9):1059-1068. DOI
10.1134/S1022795418090053

Coleman Ch.O. Karyological studies in Amphipoda (Crustacea).
Ophelia.
1994;39(2):93-105. DOI 10.1080/00785326.1994.1042
9537

Cormier A., Chebbi M.A., Giraud I., Wattier R., Teixeira M., Gilbert C.,
Rigaud T., Cordaux R. Comparative genomics of strictly vertically
transmitted, feminizing Microsporidia endosymbionts of amphipod
crustaceans. Genome Biol. Evol. 2021;13(1):evaa245. DOI 10.1093/
gbe/evaa245

Cristescu M.E., Adamowicz S.J., Vaillant J.J., Haffner D.G. Ancient
lakes revisited: from the ecology to the genetics of speciation. Mol.
Ecol. 2010;19(22):4837-4851. DOI 10.1111/j.1365-294X.2010.
04832.x

Daneliya M.E., Kamaltynov R.M., Kontula T., Väinölä R. Systematics
of the Baikalian Babr (Crustacea: Amphipoda: Pallaseidae). Zootaxa.
2009;2276(1):49-68. DOI 10.11646/zootaxa.2276.1.3

Daneliya M.E., Kamaltynov R.M., Väinölä R. Phylogeography and
systematics of Acanthogammarus s. str., giant amphipod crustaceans
from Lake Baikal. Zool. Scr. 2011;40(6):623-637. DOI 10.1111/
j.1463-6409.2011.00490.x

Drozdova P., Kizenko A., Saranchina A., Gurkov A., Firulyova M.,
Govorukhina E., Timofeyev M. The diversity of opsins in Lake Baikal
amphipods (Amphipoda: Gammaridae). BMC Ecol. Evol. 2021;
21(1):81. DOI 10.1186/s12862-021-01806-9

Drozdova P., Saranchina A., Madyarova E., Gurkov A., Timofeyev M.
Experimental crossing confirms reproductive isolation between
cryptic species within Eulimnogammarus verrucosus (Crustacea:
Amphipoda) from Lake Baikal. Int. J. Mol. Sci. 2022;23(18):10858.
DOI 10.3390/ijms231810858

Drozdova P.B., Saranchina A.E., Mutin A.D., Rzhechitskiy Ya.A.,
Gurkov A.N., Lipaeva P.V., Shatilina Zh.M., Timofeyev M.A. Geographic
barriers and reproductive isolation in the formation of crypic
species within the abundant representatives of Baikal endemic amphipods
of the genus Eulimnogammarus. In: Proceedings of the
IV All-Russia Conference “Development of Life on Earth in Abiotic Change Processes”, 25–29 Sept. 2023, Listvyanka. Irkutsk, 2023;
70-73. DOI 10.24412/cl-34446-2023-4-70-73 (in Russian)

Eberle J., Ahrens D., Mayer C., Niehuis O., Misof B. A plea for standardized
nuclear markers in Metazoan DNA taxonomy. Trends Ecol.
Evol. 2020;35(4):336-345. DOI 10.1016/j.tree.2019.12.003

Eme D., Westfall K.M., Matthíasardóttir B., Kristjánsson B.K., Pálsson
S. Contrasting phylogeographic patterns of mitochondrial and
genome-wide variation in the groundwater amphipod Crangonyx
islandicus that survived the Ice Age in Iceland. Diversity. 2023;
15(1):88. DOI 10.3390/d15010088

Fazalova V., Nevado B., Peretolchina T., Petunina J., Sherbakov D.
When environmental changes do not cause geographic separation of
fauna: differential responses of Baikalian invertebrates. BMC Evol.
Biol. 2010;10(1):320. DOI 10.1186/1471-2148-10-320

Fišer C., Robinson C.T., Malard F. Cryptic species as a window into the
paradigm shift of the species concept. Mol. Ecol. 2018;27(3):613-
635. DOI 10.1111/mec.14486

Folmer O., Black M., Hoeh W., Lutz R., Vrijenhoek R. DNA primers
for amplification of mitochondrial cytochrome c oxidase subunit I
from diverse metazoan invertebrates. Mol. Mar. Biol. Biotechnol.
1994;3(5):294-299.

Gomanenko G.V., Kamaltynov R.M., Kuzmenkova Zh.V., Berenos K.,
Sherbakov D.Yu. Population structure of the Baikalian amphipod
Gmelinoides fasciatus (Stebbing). Russ. J. Genet. 2005;41(8):907-
912. DOI 10.1007/s11177-005-0179-5

Gregory T.R., Nicol J.A., Tamm H., Kullman B., Kullman K., Leitch I.J.,
Murray B.G., Kapraun D.F., Greilhuber J., Bennett M.D. Eukaryotic
genome size databases. Nucleic Acids Res. 2007;35(Suppl.1):D332-
D338. DOI 10.1093/nar/gkl828

Gurkov A., Rivarola-Duarte L., Bedulina D., Fernández Casas I., Michael
H., Drozdova P., Nazarova A., Govorukhina E., Timofeyev M.,
Stadler P.F., Luckenbach T. Indication of ongoing amphipod speciation
in Lake Baikal by genetic structures within endemic species.
BMC Evol. Biol. 2019;19(1):138. DOI 10.1186/s12862-019-1470-8

Hebert P.D.N., Cywinska A., Ball S.L., deWaard J.R. Biological identifications
through DNA barcodes. Proc. R. Soc. Lond. B Biol. Sci.
2003;270(1512):313-321. DOI 10.1098/rspb.2002.2218

Horton T., De Broyer C., Bellan-Santini D., Coleman C.O., Copilaș-
Ciocianu D., Corbari L., Daneliya M.E., Dauvin J.-C., Decock W.,
Fanini L., Fišer C., Gasca R., Grabowski M., Guerra-García J.M.,
Hendrycks E.A., Hughes L.E., Jaume D., Kim Y.-H., King R.A.,
Lo Brutto S., Lörz A.-N., Mamos T., Serejo C.S., Senna A.R., Souza-
Filho J.F., Tandberg A.H.S., Thurston M.H., Vader W., Väinölä R.,
Valls Domedel G., Vandepitte L., Vanhoorne B., Vonk R.,
White K.N., Zeidler W. The World Amphipoda Database: history
and progress. Rec. Aust. Mus. 2023;75(4):329-342. DOI 10.3853/
j.2201-4349.75.2023.1875

Hou Z., Sket B., Li S. Phylogenetic analyses of Gammaridae crustacean
reveal different diversification patterns among sister lineages in
the Tethyan region. Cladistics. 2014;30(4):352-365. DOI 10.1111/
cla.12055

Hultgren K.M., Jeffery N.W., Moran A., Gregory T.R. Latitudinal variation
in genome size in crustaceans. Biol. J. Linn. Soc. 2018;123(2):
348-359. DOI 10.1093/biolinnean/blx153

Jeffery N.W., Yampolsky L., Gregory T.R. Nuclear DNA content correlates
with depth, body size, and diversification rate in amphipod
crustaceans from ancient Lake Baikal, Russia. Genome. 2017;60(4):
303-309. DOI 10.1139/gen-2016-0128

Jin S., Bian C., Jiang S., Sun S., Xu L., Xiong Y., Qiao H., Zhang W.,
You X., Li J., Gong Y., Ma B., Shi Q., Fu H. Identification of candidate
genes for the plateau adaptation of a Tibetan amphipod, Gammarus
lacustris, through integration of genome and transcriptome sequencing.
Front. Genet. 2019;10:53. DOI 10.3389/fgene.2019.00053

Jordan S., Hand B.K., Hotaling S., Delvecchia A.G., Malison R., Nissley
C., Luikart G., Stanford J.A. Genomic data reveal similar genetic
differentiation in aquifer species with different dispersal capabilities
and life histories. Biol. J. Linn. Soc. 2020;129(2):315-322. DOI
10.1093/biolinnean/blz173

Jourdan J., Bundschuh M., Copilaș-Ciocianu D., Fišer C., Grabowski
M., Hupało K., Jemec Kokalj A., Kabus J., Römbke J., Soose L.J.,
Oehlmann J. Cryptic species in ecotoxicology. Environ. Toxicol.
Chem. 2023;42(9):1889-1914. DOI 10.1002/etc.5696

Kamaltynov R.M. Amphipods (Amphipoda: Gammaroidea). In: Index
of Animal Species Inhabiting Lake Baikal and its Catchment Area.
Novosibirsk, 2001;I(1):572-831 (in Russian)

Kamaltynov R.M. Higher crustaceans (Amphipoda: Gammaroidea) of
Angara and Yenisey. In: Index of Animal Species Inhabiting Lake
Baikal and its Catchment Area. Novosibirsk, 2009;II(1):297-329 (in
Russian)]

Kao D., Lai A.G., Stamataki E., Rosic S., Konstantinides N., Jarvis E.,
Di Donfrancesco A., Pouchkina-Stancheva N., Sémon M., Grillo M.,
Bruce H., Kumar S., Siwanowicz I., Le A., Lemire A., Eisen M.B.,
Extavour C., Browne W.E., Wolff C., Averof M., Patel N.H., Sarkies
P., Pavlopoulos A., Aboobaker A. The genome of the crustacean
Parhyale hawaiensis, a model for animal development, regeneration,
immunity and lignocellulose digestion. eLife. 2016;5:e20062.
DOI 10.7554/eLife.20062

Kovalenkova M.V. Analysis of the Evolution of Species-rich Groups of
Baikal Invertebrates Based on Intron Sequences of ATP Synthase α-
and β-subunit Genes. PhD Thesis. Irkutsk, 2018 (in Russian)

Lavrov D.V., Pett W. Animal mitochondrial DNA as we do not know
it: mt-genome organization and evolution in nonbilaterian lineages.
Genome Biol. Evol. 2016;8(9):2896-2913. DOI 10.1093/gbe/evw195

Lefébure T., Douady C.J., Gouy M., Gibert J. Relationship between
morphological taxonomy and molecular divergence within Crustacea:
proposal of a molecular threshold to help species delimitation.
Mol. Phylogenet. Evol. 2006;40(2):435-447. DOI 10.1016/j.ympev.
2006.03.014

Macdonald K.S. III, Yampolsky L., Duffy J.E. Molecular and morphological
evolution of the amphipod radiation of Lake Baikal. Mol.
Phylogenet. Evol. 2005;35(2):323-343. DOI 10.1016/j.ympev. 2005.
01.013

Mamos T., Grabowski M., Rewicz T., Bojko J., Strapagiel D., Burzyński
A. Mitochondrial genomes, phylogenetic associations, and SNP
recovery for the key invasive Ponto-Caspian amphipods in Europe.
Int. J. Mol. Sci. 2021;22(19):10300. DOI 10.3390/ijms221910300

Mashiko K., Kamaltynov R., Morino H., Sherbakov D.Y. Genetic differentiation
among gammarid (Eulimnogammarus cyaneus) populations
in Lake Baikal, East Siberia. Arch. Hydrobiol. 2000;148(2):
249-261. DOI 10.1127/archiv-hydrobiol/148/2000/249

Mats V.D., Shcherbakov D.Y., Efimova I.M. Late Cretaceous–Cenozoic
history of the Lake Baikal depression and formation of its unique
biodiversity. Stratigr. Geol. Correl. 2011;19(4):404-423. DOI
10.1134/S0869593811040058

Moskalenko V.N., Neretina T.V., Yampolsky L.Y. To the origin of
Lake Baikal endemic gammarid radiations, with description of two
new Eulimnogammarus spp. Zootaxa. 2020;4766(3):457-471. DOI
10.11646/zootaxa.4766.3.5

Natyaganova A.V., Sitnikova T.Y. Karyotype of the Baikal amphipod
Polyacanthisca calceolata Bazikalova, 1937, (Crustacea, Amphipoda).
Chromosome Sci. 2012;15(1-2):43-48. DOI 10.11352/scr.
15.43

Naumenko S.A., Logacheva M.D., Popova N.V., Klepikova A.V.,
Penin A.A., Bazykin G.A., Etingova A.E., Mugue N.S., Kondrashov
A.S., Yampolsky L.Y. Transcriptome-based phylogeny of endemic
Lake Baikal amphipod species flock: fast speciation accompanied by frequent episodes of positive selection. Mol. Ecol. 2017;
26(2):536-553. DOI 10.1111/mec.13927

Patra A.K., Chung O., Yoo J.Y., Kim M.S., Yoon M.G., Choi J.-H.,
Yang Y. First draft genome for the sand-hopper Trinorchestia longiramus.
Sci. Data. 2020;7(1):85. DOI 10.1038/s41597-020-0424-8

Patra A.K., Chung O., Yoo J.Y., Baek S.H., Jung T.W., Kim M.S.,
Yoon M.G., Yang Y., Choi J.-H. The draft genome sequence of
a new land-hopper Platorchestia hallaensis. Front. Genet. 2021;11:
621301. DOI 10.3389/fgene.2020.621301

Petunina Z.V. Comparative Ecological and Genetic Analysis of Microsporidia
and Their Host, the Baikal Amphipod Gmelinoides fasciatus.
PhD Thesis. Irkutsk, 2015 (in Russian)

Petunina J.V., Vavrishchuk N.V., Romanova E.V. Variability of morphological
and genetic traits of Macrohectopus branickii. In: Development
of Physical and Chemical Biology, Bioengineering and
Bioinformatics at the Present Stage: Abstracts of reports of the
IV All-Russian sci. and pract. conf. with int. participation, dedicated
to the 45th anniversary of the Department of Physical and Chemical
Biology, Bioengineering and Bioinformatics of ISU. Irkutsk, October
25–27, 2023. Irkutsk: Irkutsk State University Publ., 2023;111-
113 (in Russian)

Poynton H.C., Hasenbein S., Benoit J.B., Sepulveda M.S., Poelchau
M.F., Hughes D.S.T., Murali S.C., Chen S., Glastad K.M.,
Goodisman M.A.D., … Dinh H., Han Y., Doddapaneni H., Worley
K.C., Muzny D.M., Gibbs R.A., Richards S. The toxicogenome
of Hyalella azteca: a model for sediment ecotoxicology and evolutionary
toxicology. Environ. Sci. Technol. 2018;52(10):6009-6022.
DOI 10.1021/acs.est.8b00837

Ratnasingham S., Hebert P.D.N. BOLD: The Barcode of Life Data System
(http://www.barcodinglife.org). Mol. Ecol. Notes. 2007;7(3):
355-364. DOI 10.1111/j.1471-8286.2007.01678.x

Ratnasingham S., Hebert P.D.N. A DNA-based registry for all animal
species: The Barcode Index Number (BIN) System. PLoS One.
2013;8(7):e66213. DOI 10.1371/journal.pone.0066213

Rivarola-Duarte L. Unraveling the genetic secrets of ancient Baikal
amphipods. PhD Thesis. Leipzig: Universität Leipzig, 2021

Rivarola-Duarte L., Otto C., Jühling F., Schreiber S., Bedulina D.,
Jakob L., Gurkov A., Axenov-Gribanov D., Sahyoun A.H., Lucassen
M., Hackermüller J., Hoffmann S., Sartoris F., Pörtner H.-O.,
Timofeyev M., Luckenbach T., Stadler P.F. A first glimpse at the
genome of the Baikalian amphipod Eulimnogammarus verrucosus.
J. Exp. Zoolog. B Mol. Dev. Evol. 2014;322(3):177-189. DOI
10.1002/jez.b.22560

Romanova E.V., Aleoshin V.V., Kamaltynov R.M., Mikhailov K.V.,
Logacheva M.D., Sirotinina E.A., Gornov A.Yu., Anikin A.S.,
Sherbakov D.Yu. Evolution of mitochondrial genomes in Baikalian
amphipods. BMC Genomics. 2016a;17(14):1016. DOI 10.1186/
s12864-016-3357-z

Romanova E.V., Mikhailov K.V., Logacheva M.D., Kamaltynov R.M.,
Aleoshin V.V., Sherbakov D.Y. The complete mitochondrial genome
of Baikalian amphipoda Eulimnogammarus vittatus (Dybowsky,
1874). Mitochondrial DNA Part A. 2016b;27(3):1795-1797. DOI
10.3109/19401736.2014.963817

Romanova E.V., Mikhailov K.V., Logacheva M.D., Kamaltynov R.M.,
Aleoshin V.V., Sherbakov D.Yu. The complete mitochondrial genome
of a deep-water Baikalian amphipoda Brachyuropus grewingkii
(Dybowsky, 1874). Mitochondrial DNA Part A. 2016c;27(6):
4158-4159. DOI 10.3109/19401736.2014.1003891

Romanova E.V., Bukin Y.S., Mikhailov K.V., Logacheva M.D.,
Aleoshin V.V., Sherbakov D.Yu. Hidden cases of tRNA gene duplication
and remolding in mitochondrial genomes of amphipods. Mol.
Phylogenet. Evol. 2020;144:106710. DOI 10.1016/j.ympev.2019.
106710

Romanova E.V., Bukin Y.S., Mikhailov K.V., Logacheva M.D.,
Aleoshin V.V., Sherbakov D.Y. The mitochondrial genome of
a freshwater pelagic amphipod Macrohectopus branickii is among
the longest in Metazoa. Genes. 2021;12(12):2030. DOI 10.3390/
genes12122030

Salemaa H., Kamaltynov R.M. The chromosome numbers of endemic
Amphipoda and Isopoda – an evolutionary paradox in the ancient
lakes Ohrid and Baikal. In: Martens K., Goddeeris B., Coulter G.
(Eds.) Speciation in Ancient Lakes. Advances in Limnology. Vol. 44.
Stuttgart (Germany): Schweizerbart Science Publ.,1994;247-256

Shao C., Sun S., Liu K., Wang J., Li S., Liu Q., Deagle B.E., Seim I.,
Biscontin A., Wang Q., … Zhang G., Yang H., Xu X., Wang J.,
Zhao X., Meyer B., Fan G. The enormous repetitive Antarctic krill
genome reveals environmental adaptations and population insights.
Cell. 2023;186(6):1279-1294.e19. DOI 10.1016/j.cell.2023.02.005

Sherbakov D.Y. Molecular phylogenetic studies on the origin of biodiversity
in Lake Baikal. Trends Ecol. Evol. 1999;14(3):92-95. DOI
10.1016/S0169-5347(98)01543-2

Sherbakov D.Yu., Kovalenkova M.V., Maikova O.O. Some results of
molecular phylogenetic studies of Baikal endemic invertebrates.
Russ. J. Genet. Appl. Res. 2017;7(4):345-349. DOI 10.1134/S20790
59717040104

Sket B., Morino H., Takhteev V., Rogers D.C. Chapter 16.6 – Phylum
Arthropoda: Malacostraca. In: Thorp and Covich’s Freshwater Invertebrates.
Vol. 4: Keys to Palaearctic Fauna. Boston: Acad. Press,
2019;789-889. DOI 10.1016/B978-0-12-385024-9.00022-8

Smith D.R. The past, present and future of mitochondrial genomics:
have we sequenced enough mtDNAs? Brief. Funct. Genomics.
2016;15(1):47-54. DOI 10.1093/bfgp/elv027

Takhteev V.V. Essays on the Amphipods of Lake Baikal (Systematics,
comparative ecology, evolution). Irkutsk, 2000a (in Russian)

Takhteev V.V. Trends in the evolution of Baikal amphipods and evolutionary
parallels with some marine malacostracan faunas. In: Advances
in Ecological Research. Vol. 31: Ancient Lakes: Biodiversity,
Ecology and Evolution. Acad. Press, 2000b;197-220. DOI 10.1016/
S0065-2504(00)31013-3

Takhteev V. On the current state of taxonomy of the Baikal Lake amphipods
(Crustacea, Amphipoda) and the typological ways of constructing
their system. Arthropoda Sel. 2019;28(1):374-402. DOI
10.15298/arthsel.28.3.03

Takhteev V.V., Berezina N.A., Sidorov D.A. Checklist of the Amphipoda
(Crustacea) from continental waters of Russia, with data on
alien species. Arthropoda Sel. 2015;24(3):335-370. DOI 10.15298/
arthsel.24.3.09

Toews D.P.L., Brelsford A. The biogeography of mitochondrial and
nuclear discordance in animals. Mol. Ecol. 2012;21(16):3907-3930.
DOI 10.1111/j.1365-294X.2012.05664.x

Väinölä R., Kamaltynov R.M. Species diversity and speciation in the
endemic amphipods of Lake Baikal: molecular evidence. Crustaceana.
1999;72(8):945-956

Weston J.N.J., Jensen E.L., Hasoon M.S.R., Kitson J.J.N., Stewart
H.A., Jamieson A.J. Barriers to gene flow in the deepest ocean
ecosystems: evidence from global population genomics of a cosmopolitan
amphipod. Sci. Adv. 2022;8(43):eabo6672. DOI 10.1126/
sciadv.abo6672

Wyngaard G.A., Skern-Mauritzen R., Malde K., Prendergast R., Peruzzi
S. The salmon louse genome may be much larger than sequencing
suggests. Sci. Rep. 2022;12(1):6616. DOI 10.1038/s41598-022-
10585-2

Yampolsky L.Yu., Kamaltynov R.M., Ebert D., Filatov D.A., Chernykh
V.I. Variation of allozyme loci in endemic gammarids of Lake
Baikal. Biol. J. Linn. Soc. 1994;53(4):309-323. DOI 10.1111/j.1095-
8312.1994.tb01015.x

Yuxiang W., Peretolchina T.E., Romanova E.V., Sherbakov D.Y. Comparison
of the evolutionary patterns of DNA repeats in ancient and
young invertebrate species flocks of Lake Baikal. Vavilov J. Genet.
Breed. 2023;27(4):349-356. DOI 10.18699/VJGB-23-42

Zaidykov I.Y., Naumova E.Y., Sukhanova L.V. MtDNA polymorphism
of Macrohectopus branickii Dybowsky, 1974 (Amphipoda) – an endemic
pelagic key species of Lake Baikal. In: Chaplina T. (Ed.) Complex
Investigation of the World Ocean (CIWO-2023). Springer Nature
Switzerland, 2023;223-229. DOI 10.1007/978-3-031-47851-2_26

Zolotovskaya E., Nazarova A., Saranchina A., Mutin A., Drozdova P.,
Lubyaga Y., Timofeyev M. Hemocyte proteome of the Lake Baikal
endemic Eulimnogammarus verrucosus (Crustacea: Amphipoda)
sheds light on immune-related proteins. Biol. Commun. 2021;66(4):
290-301. DOI 10.21638/spbu03.2021.402

